# Phenotypic and functional alterations of monocyte subsets with aging

**DOI:** 10.1186/s12979-022-00321-9

**Published:** 2022-12-13

**Authors:** Yu Cao, Yang Fan, Fangyuan Li, Yu Hao, Yaxian Kong, Chen Chen, Xing Hao, Dannuo Han, Guoli Li, Zengtao Wang, Chuan Song, Junyan Han, Hui Zeng

**Affiliations:** 1 Beijing Key Laboratory of Emerging Infectious Diseases, Institute of Infectious Diseases, Beijing Ditan Hospital, Capital Medical University, Beijing, 100015 China; 2 Beijing Institute of Infectious Diseases, Beijing, 100015 China; 3 National Center for Infectious Diseases, Beijing Ditan Hospital, Capital Medical University, Beijing, 100015 China; 4 Biomedical Innovation Center, Beijing Shijitan Hospital, Capital Medical University, Beijing, 100038 China; 5 Beijing Key Laboratory for Therapeutic Cancer Vaccines, Beijing Shijitan Hospital, Capital Medical University, Beijing, 100038 China; 6 Center for Cardiac Intensive Care, Beijing Anzhen Hospital, Capital Medical University, Beijing, 100029 China

**Keywords:** Aging, Monocytes, Immunosenescence, Activation

## Abstract

**Background:**

It has been widely accepted that monocytes are one of the central mediators contributing to inflammaging. However, it remains unclear whether aged monocytes, similar to aged T cells, have characteristics of hyperactivation and increased expression of co-inhibitory molecules.

**Methods:**

Peripheral blood mononuclear cells (PBMCs) were isolated from young (21–40 years old), middle-aged (41–60 years old), and older human subjects (> 60 years old). Flow cytometry was used to monitor changes in the expression of surface molecules of monocyte subsets and cytokine-producing capacity.

**Results:**

We observed increased tumor necrosis factor-α: TNF-α and decreased interleukin-6 (IL-6) production in monocytes from older adults compared with young and middle-aged adults. Older adults had a greater percentage of intermediate and non-classical monocyte subsets, along with increased levels of the immune activation markers human leukocyte antigen-DR (HLA-DR), and adhesion molecules cluster of differentiation molecule 11b (CD11b) and L-selectin (CD62L). Furthermore, we observed increased C–C motif chemokine receptor 2 (CCR2) expression on classical monocytes and decreased C-X3-C motif chemokine receptor 1 (CX3CR1) expression on non-classical monocytes in older adult subjects. The expression of co-inhibitory receptors was reduced on monocyte subsets in older adults.

**Conclusions:**

Circulating monocytes in older adults exhibit increased expression of activation, adhesion, and migration markers, but decreased expression of co-inhibitory molecules.

**Supplementary Information:**

The online version contains supplementary material available at 10.1186/s12979-022-00321-9.

## Background

With the increase in worldwide life expectancy, the percentage of older people in the general population has grown dramatically [1]. The immune system is one physiological system undergoing dramatic changes during aging. Characteristic aging-associated changes have been described for various aspects of the immune system in terms of numbers, phenotypes, and functions [2].

As one of the central phenotypes associated with aging, chronic immune activation causes two distinct but closely interrelated aspects of aging: immunosenescence and inflammaging [3, 4]. Immunosenescence refers to the age-related decline in immune functions, such as thymic involution and dampened responses to infections or vaccinations [5]. Ultimately, immunosenescence leads to a decreased capacity to combat infections and poor vaccine efficacy in the elderly [6]. In contrast, inflammaging displays a chronic, sterile, low-grade, and age-related inflammation accompanied by chronic activation of the immune system, and thus contributes to the pathogenesis of various age-related diseases [7]. In addition, inflammaging may lead to an over-exuberant immune response to viral infections, which further causes more severe tissue injury and enhances mortality in the elderly [8, 9].

An accumulation of certain cell populations with the characteristics of immunosenescence and inflammaging is a conserved hallmark of immune aging. For instance, the elderly have an age-related accumulation of PD-1^+^TIGIT^+^CD8^+^ exhausted T cells [10] and granzyme K-expressing CD8^+^ T cell populations with an inflammaging phenotype [11]. Aged immune cells can also exhibit features of both immunosenescence and inflammaging. For example, CD8^+^ T cells from the elderly display an exhaustion phenotype (upregulation of co-inhibitory receptors, impaired cytokine production, and high susceptibility to apoptosis) [12], as well as higher levels of intracellular Granzyme B and perforin [13].

Monocytes, the precursor of macrophages [14], play a crucial role in innate immune responses [15, 16]. Circulating monocytes are heterogeneous [17] and consist of three subsets: CD14^high^CD16^−^ classical monocytes, CD14^high^CD16^+^ intermediate monocytes, and CD14^low^CD16^+^ non-classical monocytes [18–20]. Additionally, we reported a CD14^+/low^CD16^−^ monocyte subset that represents immature monocytes newly released from the bone marrow [21]. Among these populations, CD14^high^CD16^+^ intermediate monocytes exhibit a high capacity for the production of tumor necrosis factor-α (TNF-α) and regulating immune responses [20] and closely correlate with many inflammatory diseases. CD14^low^CD16^+^ monocytes express higher levels of the C-X3-C motif chemokine receptor 1 (CX3CR1), patrol the vascular endothelium, and are involved in the immune surveillance of local tissues [22]. It has been widely accepted that monocytes are one of the central components of inflammaging [5]. Several recent studies have reported that elderly individuals have altered proportions of monocyte subsets [23] correlating with an increased incidence of chronic inflammatory diseases [24–26]. In addition, although monocytes and macrophages from older individuals exhibit impaired phagocytosis capabilities, higher intracellular TNF-α [27] was detected at basal levels and upon stimulation with toll-like receptor (TLR) 4 or TLR1/2 agonists [28]. Cumulatively, these findings support the notion that monocyte populations may facilitate the inflammaging process.

Previous studies have revealed a crucial role of co-inhibitory molecules in T cell aging [10–13]. Recently, co-inhibitory molecules have drawn considerable attention in the innate immune system. It has been shown that co-inhibitory molecules may suppress innate immune responses and promote immune tolerance [29, 30]. As one of the major compartments of the innate immune system, monocytes have been widely accepted as central mediators of inflammaging [15]. However, phenotypic and functional alterations in monocyte subsets have not been comprehensively investigated. Specifically, it remains unclear whether aged monocytes have characteristics similar to aged T cells, such as increased expression of co-inhibitory molecules and hyperactivation phenotypes. Herein, we demonstrated that circulating monocytes in older adults exhibit increased markers of activation, adhesion, and migration, but decreased expression of co-inhibitory molecules.

## Results

### Increased TNF-α and decreased IL-6 production by aging monocytes

In the present study, 192 healthy adults (males: 75; females: 117) were recruited and subdivided into three groups: young (21–40 years old), middle-aged (41–60 years old), and older adults (> 60 years old) (Table 1). A chi-squared test was performed and demonstrated that gender was balanced among all three groups (*P* = 0.6860).

**Table 1:** Characteristics of adult study participants

Parameters	Total (*n* = 192)	21–40 (*n* = 65)	41–60 (*n* = 49)	> 60 (*n* = 78)	*P* Value
Gender					
Male	75	33	17	25	0.6860
Female	117	32	32	53	
Age, years					
Median	51.5	33	49.5	73	0.0014
IQR	36–69	28–36	43–55	65.5–78	

Age was described by the median and interquartile range (IQR) and analyzed using the Kruskal–Wallis test

**Fig. 1:**
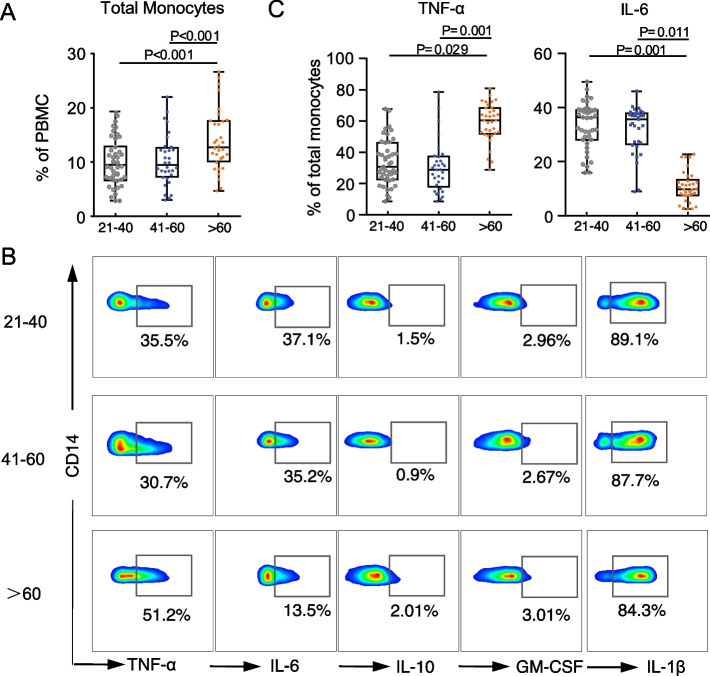
The cytokine profiles of total monocytes in healthy adults in different age groups. **A**. Comparison of the percentage of total monocytes among peripheral blood mononuclear cells (PBMCs) in young, middle-aged, and older adults via flow cytometry (young: 21–40 years, *n* = 42; middle-aged: 41–60 years, *n* = 34; older: > 60 years, *n* = 34). Data are shown as a box plot with medians (lines inside boxes), 25^th^, and 75^th^ quartiles (limits of boxes). Whiskers indicate the range, and each dot represents one sample. P-values were obtained using a Kruskal–Wallis test, followed by post hoc analysis. **B**. Representative flow cytometry data of intracellular staining for TNF-α, IL-6, IL-10, GM-CSF, and IL-1β in total monocytes stimulated with LPS (100 ng/mL) for 3 h in vitro from young, middle-aged, and older adults (young: 21–40 years, *n* = 42; middle-aged: 41–60 years, *n* = 34; older: > 60 years, *n* = 34). **C**. Intracellular staining for the percentage of TNF-α^+^ and IL-6^+^ monocytes stimulated with LPS (100 ng/mL) for 3 h in vitro from young, middle-aged, and older adults (young: 21–40 years, *n* = 42; middle-aged: 41–60 years, *n* = 34; older: > 60 years, *n* = 34) upon in vitro LPS stimulation. Data are shown as box-plots with medians (lines inside boxes), 25^th^, and 75^th^ quartiles (limits of boxes). Whiskers indicate the range, and each dot represents one sample. P-values were obtained by a Kruskal–Wallis rank test, followed by post hoc analysis

Older adults exhibited a significant increase in the percentage of total monocytes in PBMCs and in the number of total monocytes in whole blood than adults in the young and middle-aged groups (Fig. 1A; Fig. S1B). At baseline (mock control), the percentages of TNF-α^+^, IL-6^+^, interleukin-10 (IL-10)^+^, granulocyte–macrophage colony-stimulating factor (GM-CSF)^+^, and interleukin-1β (IL-1β)^+^ monocytes were comparable among all three groups (Fig. S2A, S2B). In vitro LPS-stimulated total monocytes from elderly participants had a higher frequency of TNF-α^+^ cells and increased TNF-α:expression based on median fluorescence intensity (MFI) (Fig. 1B; 1C; S3B) compared to middle-aged and young adults. Further, LPS-stimulated total monocytes in PBMCs from elderly participants had a lower percentage of IL-6^+^ monocytes and reduced IL-6 expression than those from middle-aged and young adults (Fig. 1B; 1C; S3B). Lastly, the frequency and expression of IL-10, GM-CSF, and IL-1β: in total monocytes were comparable among all three age groups (Fig. 1B; S3A; S3B).

### Increased intermediate and non-classical monocyte subsets in older adults

**Fig. 2:**
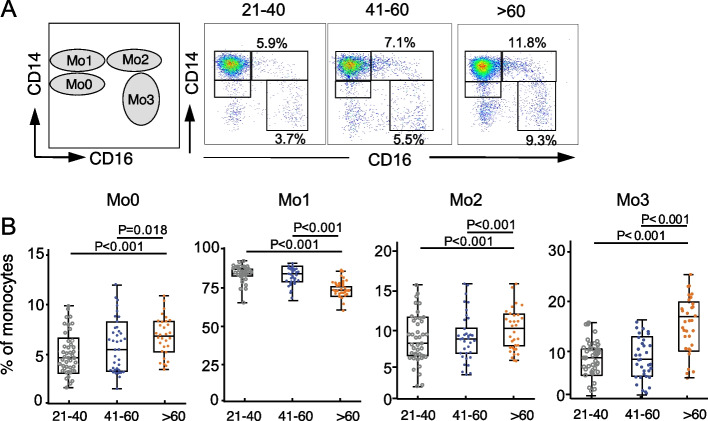
Percentage of peripheral monocyte subsets from young, middle-aged, and older adults. **A**. Ideograph and representative flow cytometry analyses of monocytes. According to the expression pattern of CD14 and CD16, human monocytes were divided into CD14^low^CD16^−^ (Mo0), CD14^high^CD16^−^ (Mo1), CD14^high^CD16^+^ (Mo2), and CD14^low^ CD16^+^ (Mo3) subsets. **B**. Comparison of the percentages of Mo0, Mo1, Mo2, and Mo3 subpopulations among all monocytes in young, middle-aged, and older adults were performed according to the expression pattern of CD14 and CD16 (young: 21–40 years, *n* = 42; middle-aged: 41–60 years, *n* = 34; older: > 60 years, *n* = 34). P-values were calculated using the non-parametric Kruskal–Wallis rank test, followed by post hoc analysis

According to previous studies, circulating monocytes are schematically divided into four subsets: immature CD14^low^CD16^−^ phenotype (Mo0), classical CD14^high^CD16^−^ (Mo1), intermediate CD14^high^CD16^+^ (Mo2), and non-classical CD14^low^CD16^+^ (Mo3) subsets (Fig. 2A; S1A). In our study, older adults exhibited decreased frequency of Mo1 monocytes and increased frequencies and numbers of Mo0, Mo2 and Mo3 monocyte subsets compared to young and middle-aged adults (Fig. 2B; S1C). Cumulatively, these data indicated that a shift in monocyte subsets to intermediate and non-classical phenotypes occurred during the aging process.

### Expression of activation, adhesion, and chemokine receptor markers on monocyte subsets

**Fig. 3:**
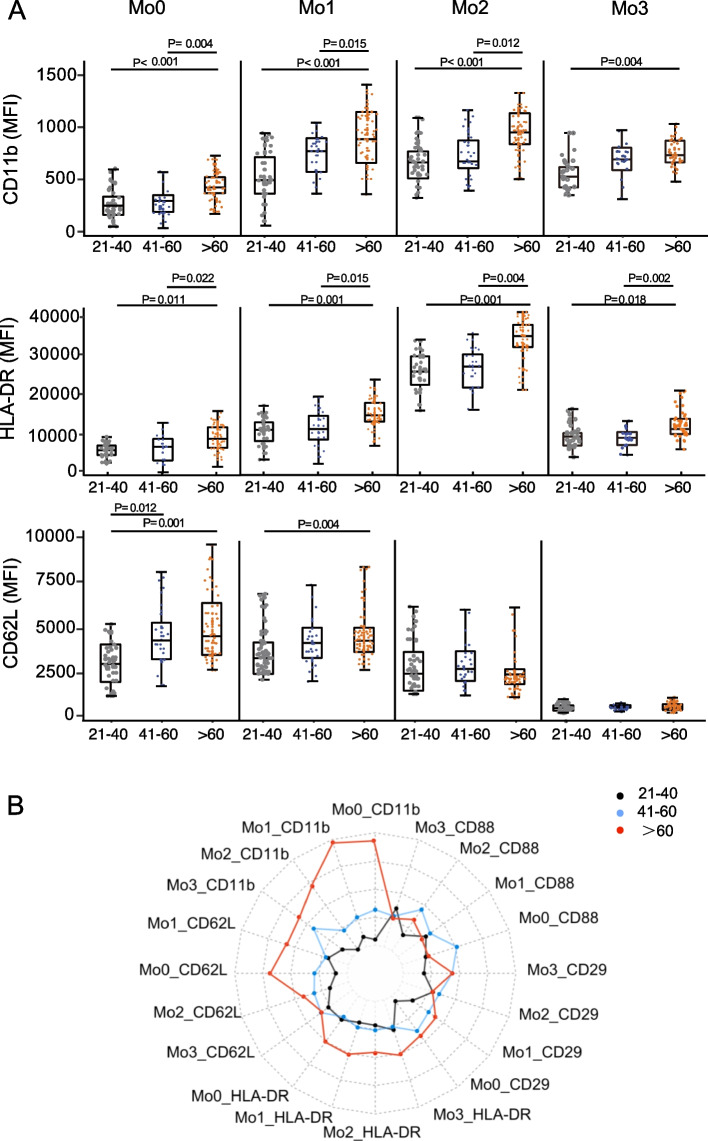
The expression levels of HLA-DR, CD88, CD29, CD11b, and CD62L in monocyte subsets. **A**. The MFI of HLA-DR, CD11b, and CD62L from monocyte subsets (Mo0, Mo1, Mo2, and Mo3) among young, middle-aged, and older persons (young: 21–40 years, *n* = 23; middle-aged: 41–60 years, *n* = 15; older: > 60 years, *n* = 44). **B**. The Z-scores of HLA-DR, CD88, CD29, CD11b, and CD62L expression of monocyte subsets (Mo0, Mo1, Mo2, and Mo3) from young (black dots, *n* = 23), middle-aged (blue dots, *n* = 15) and older adults (red dots, *n* = 44) as a radar plot, and the values range from 0 to 1

We next investigated the expression of activation molecules (HLA-DR and CD88), and adhesion molecules (CD29, CD11b, and CD62L) on different monocyte subsets (Table S1; Fig. S4A; S4B). Compared to young adults, elderly individuals displayed a marked increase in CD11b expression levels on all monocyte subsets (Fig. 3A; S5A). Similarly, the expression of HLA-DR on all monocyte subsets from elderly individuals was higher than that of monocytes from young and middle-aged adults (Fig. 3A; S5A). Additionally, Mo1 and Mo0 subsets from elderly adults expressed increased levels of CD62L (Fig. 3A; S5A) and increased CD29 expression levels on Mo0 monocytes compared with those from young adults (Fig. S5A; S5B). CD88 expression levels were comparable among monocyte subsets regardless of age (Fig. S5A; S5B). Further, a radar graph of young, middle-aged, and older adults identified significant differences amongst normalized CD11b, CD62L, and HLA-DR levels on monocyte subsets from older adults (Fig. 3B). These data indicated that the expression levels of activation and adhesion molecules on monocytes are increased in older adults due to the aging process.

**Fig. 4:**
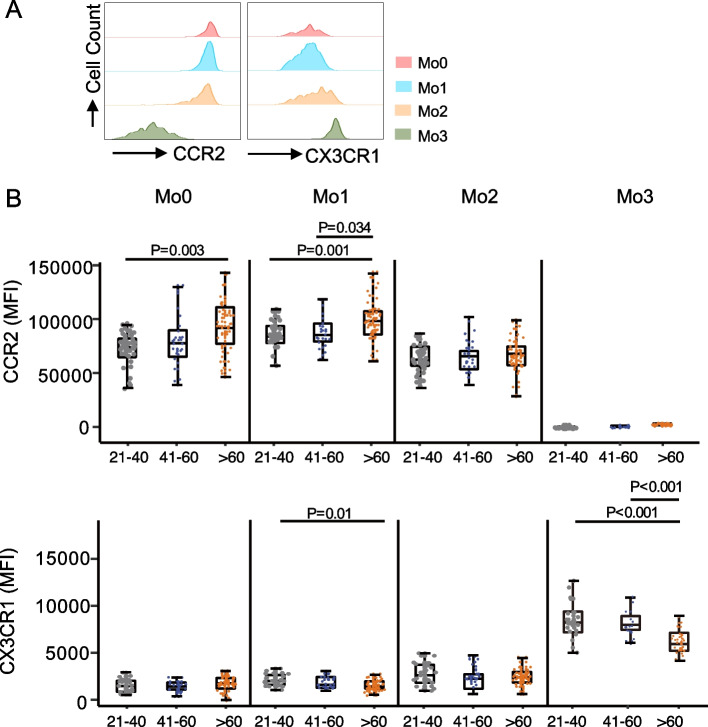
The expression of CCR2 and CX3CR1 in monocyte subsets. **A**. The MFI of CCR2 and CX3CR1 from monocyte subsets in young participants was analyzed by flow cytometry. Histograms were created using FlowJo software. **B**. Boxplots showing the MFI of CCR2 and CX3CR1 from monocyte subsets in young, middle-aged, and older individuals (young: 21–40 years, *n* = 23; middle-aged: 41–60 years, *n* = 15; older: > 60 years, *n* = 44). P-values were calculated using the non-parametric Kruskal–Wallis rank test, followed by post hoc analysis

CCR2 and CX3CR1 are two key chemokine receptors important for monocyte migration (Table S1). In conformity with previous studies [31], Mo0, Mo1, and Mo2 subsets expressed high levels of CCR2 but intermediate levels of CX3CR1, while Mo3 subsets expressed high levels of CX3CR1 (Table S1; Fig. 4A). We found increased expression of CCR2 on Mo0 and Mo1 subsets (Fig. 4B; S6A; S6B) and reduced CX3CR1 expression on Mo1 and Mo3 subsets from the elderly adult group (Fig. 4B; S6A; S6B).

### Decreased expression of co-inhibitory molecules on monocytes in the elderly

**Fig. 5:**
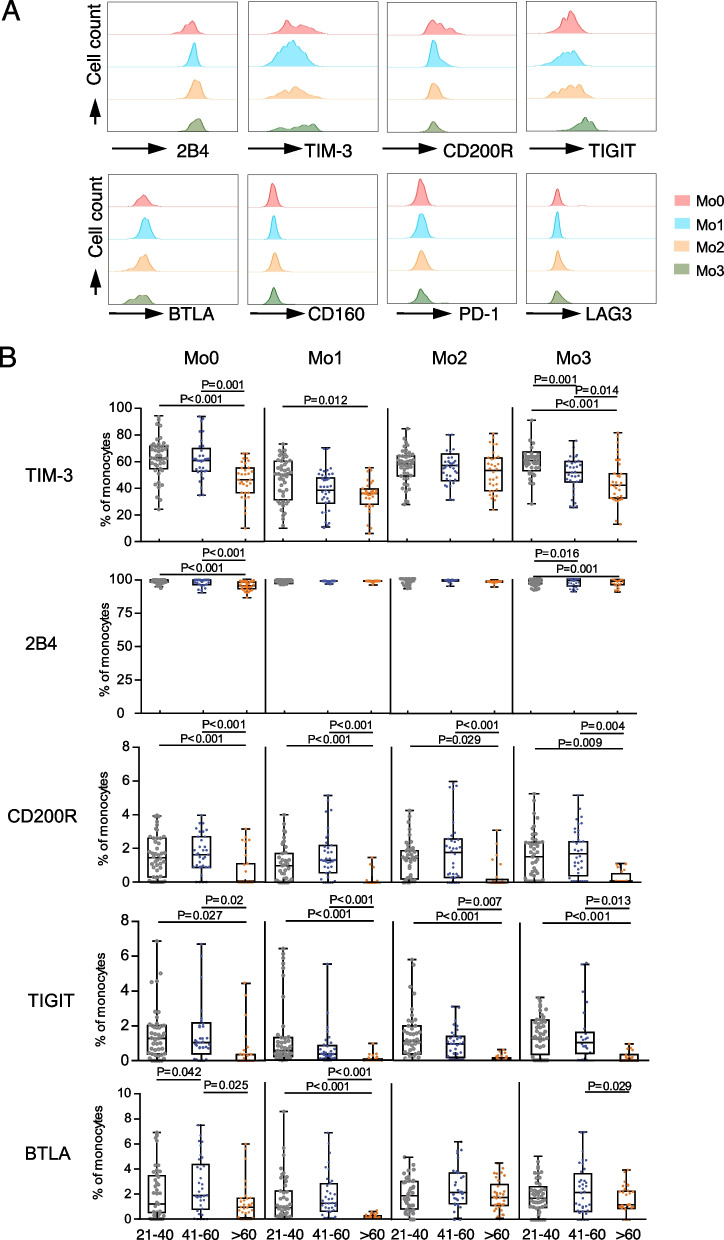
Percentage of monocyte subsets expressing co-inhibitory molecules decreased in older adults. **A**. Representative flow cytometry histograms display the expression of the co-inhibitory molecules 2B4, T-cell immunoglobulin domain and mucin domain 3 (TIM-3), CD200R, T-cell immunoglobulin and immunoreceptor tyrosine-based inhibitory motif (ITIM) domain (TIGIT), B and T lymphocyte attenuator (BTLA), CD160, programmed death-1 (PD-1), and lymphocyte-activation gene 3 (LAG-3) on monocyte subsets from young adults. **B**. The percentage of monocyte subsets expressing TIM-3, 2B4, CD200R, TIGIT, and BTLA from young, middle-aged, and older adults (young: 21–40 years, *n* = 42; middle-aged: 41–60 years, *n* = 34; older: > 60 years, *n* = 34). P-values were calculated using the non-parametric Kruskal–Wallis rank test, followed by post hoc analysis

We have previously reported that the expression of T-cell immunoglobulin and immunoreceptor tyrosine-based inhibitory motif (ITIM) domain (TIGIT) is increased in aging T cells [10]; however, whether this is true in monocyte subsets remains unknown. To test this, we measured co-inhibitory receptor expression on monocytes from all three age groups. We found that monocytes from young adults expressed 2B4 (CD244), T-cell immunoglobulin domain and mucin domain 3 (TIM-3), CD200R, TIGIT, and B and T lymphocyte attenuator (BTLA), while CD160, PD-1, and lymphocyte-activation gene 3 (LAG-3) levels were rarely expressed (Table S1; Fig. 5A). In contrast, the percentages of monocytes expressing CD200R and TIGIT were reduced on all monocyte subsets in older adults compared to young and middle-aged adults (Fig. 5B). We also observed that the Mo0, Mo1, and Mo3 subsets in elderly individuals expressed decreased levels of BTLA and TIM-3 (Fig. 5B).

## Discussion

Several studies have investigated age-related alterations in monocyte subsets in older adults [32–37]. Consistent with previous studies [38, 39], we observed an increase in the percentages of intermediate monocyte subsets with greater TNF-α producing capacity in the elderly population, indicative of inflammaging. In addition, monocytes from older adults had increased expression levels of activation markers, along with reduced expression of co-inhibitory molecules compared to monocytes from young and middle-aged adults.

Previous studies have demonstrated that both HLA-DR and co-inhibitory molecules regulate innate inflammatory responses in different manners [40–42]. Since HLA-DR crosslinking can generate intracellular signals to induce TNF-α release in monocytes, enhanced HLA-DR expression is consistent with amplified inflammatory responses in aged monocytes [43]. In contrast, co-inhibitory molecules were originally recognized as an important feature of exhausted T-cells in patients with chronic infection and cancer [10, 44]. Recent studies have demonstrated the suppressive functions and effects of co-inhibitory molecules on innate immune responses. For instance, the co-inhibitory molecule TIM-3 is expressed on monocytes, macrophages, dendritic cells (DCs), and natural killer (NK) cells [45], and impairs TNF-α:production by negatively regulating the nuclear factor kappa B (NF-κB)/TNF-α pathway [46]. The low expression levels of CD200R, 2B4, and LAG-3 correlate with increased production of TNF-α and IL-6 by monocytes [47, 48]. Therefore, co-inhibitory molecules act as negative regulators for both the innate and adaptive immune systems. Of note, T-cells and monocytes display distinct alterations during the process of aging. Long-term exposure to a wide range of antigens results in increased expression of co-inhibitory molecules in T cells, along with an impaired capacity to produce pro-inflammatory cytokines [49]. In contrast, although acute infection up-regulates the levels of co-inhibitory molecules via TLR signaling pathways in monocytes, we found that aged monocytes had a reduced expression of co-inhibitory molecules, and an increased capacity to produce TNF-α. Further studies are required to explore the underlying mechanisms regulating the unique senescence and aging features of different immune cell types.

In addition to the intermediate subset, the percentage of the non-classical monocyte subset was also increased in the circulating monocyte pool during aging. Non-classical monocytes are a typical population involved in innate immune surveillance and inflammatory responses in local tissues [50]. We found dysregulated expression of chemokine and adhesion molecules (CCR2, CX3CR1, CD11b, and CD62L) in the elderly population. Previous studies have shown the roles of these molecules in the regulation of monocyte recruitment to local tissues. CCR2 can enhance chemotactic motility and recruitment of these cells to the vessel wall [], CD62L is involved in monocyte rolling and adhesion to endothelial cells [52], CD11b mediates the trans-endothelial migration of monocytes [53,54], and CX3CR1 provides a survival signal and promotes the differentiation of non-classical monocytes into anti-inflammatory M2-like macrophages [22]. Notably, aberrant expression of these molecules can occur in other monocyte subsets beyond non-classical monocytes []. Immature Mo0 and Mo1 subsets are more susceptible to dysregulated molecules expression, indicating that immature populations may contribute to local inflammatory responses in age-associated diseases. This notion is consistent with our previous study, which demonstrated that immature CD14^low^CD16^−^ monocytes can migrate to the lungs, differentiate into mature TNF-α-producing monocytes, and contribute to acute lung injury induced by cardiopulmonary bypass [21].

Previous studies have reported elevated plasma levels of IL-6 in the elderly [55]. However, we found a decreased capacity for IL-6 production in response to LPS stimulation in total monocytes from elderly patients. It is possible that other cell types may also be a source of IL-6 in elderly individuals. This is supported by previous studies that found the elderly had lower percentages of CD14^high^CD16^−^monocytes, which have a higher capacity for IL-6 production in response to pathogens than other monocyte subsets [56, 57].

One limitation of our study is that we did not investigate the cytokine responses of each monocyte subset, due to rapid monocyte differentiation and unstable CD16 and CD14 expression of isolated cells in vitro [58]. Future clinical studies are required to confirm the correlation between the parameters and characteristics of monocyte aging and the progression of chronic age-related diseases.

## Conclusions

**Fig. 6:**
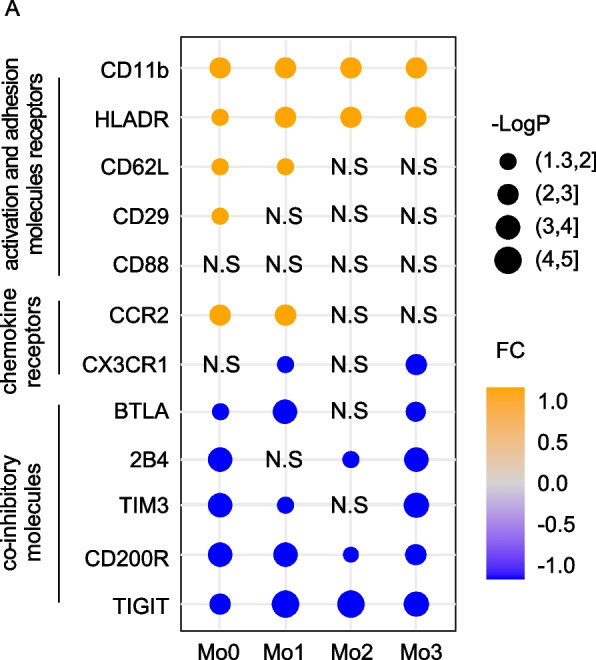
Summary of function-related molecule expression profiles of monocyte subsets between younger and older adults. The different expression levels of function-related molecules (activation and adhesion molecules and receptors, chemokine receptors, and co-inhibitory molecules) in four monocyte subsets (Mo0, Mo1, Mo2, and Mo3) from older adults (> 60 years old) compared with younger adults (< 60 years old). Fold changes were calculated by the ratio of expression levels between older and young adults. Significant differences in the molecular expression within monocyte subsets from older adults compared with young adults are presented as dots (the dot size is -LogP). *P*-values were calculated using the Wilcoxon rank-sum test. Orange and blue dots represent increased and decreased values of monocyte subsets in older adults compared to young adults

In summary, we discovered a series of alterations in the monocyte subset from elderly adults including the following: 1) increased intermediate and non-classical monocyte subsets, along with decreased classical subsets; 2) increased expression of immune activation markers, such as HLA-DR, CD11b, and CD62L by monocyte subsets; 3) increased CCR2 expression on classical monocytes and decreased CX3CR1 expression on non-classical monocytes; and 4) decreased percentage of monocytes expressing the co-inhibitory receptors 2B4, TIM-3, CD200R, TIGIT, and BTLA (Fig. 6). Further mechanistic insights and research are needed to provide a better understanding of the immune dysfunction occurring during age-related diseases, and to accelerate the development of therapeutic regimens for aging-associated inflammatory responses.

## Materials and methods

### Study subjects

A total of 192 healthy volunteers aged 24–90 years (75 males and 117 females) were recruited from September to October 2018. All human blood samples were collected after written informed consent had been obtained. The subjects who tested positive for human immunodeficiency virus (HIV) infection, hepatitis viral infections, systemic infection, connective tissue disease, cancer, or abnormal tumor markers—including alpha-fetoprotein (AFP), carcinoembryonic antigen (CEA), carbohydrate antigen (CA-199), CA-153, and CA-125—were excluded from our study. This study was approved by the committee of ethics at Beijing Ditan Hospital, Capital Medical University, Beijing, China. Similar to our previous studies [10, 44], the healthy adults were subdivided into three groups: young (21–40 years old), middle-aged (41–60 years old), and older adults (> 60 years old).

### Isolation of peripheral blood mononuclear cells (PBMCs)

Whole blood was collected in Vacutainer tubes with EDTA-K2 and processed immediately for PBMCs isolation. Blood was diluted 1:1 with phosphate-buffered saline (PBS), layered onto Ficoll-Paque (GE Healthcare, Marlborough, MA, USA), and processed according to the manufacturer’s instructions.

### Immunofluorescence staining and flow cytometric analysis

All experiments and assays were performed on freshly isolated samples. Isolated PBMCs were incubated with directly conjugated fluorescent antibodies for 30 min at 4 °C. The cells were washed before flow cytometry analysis. Monocytes were separated from other cells by gating on CD3/15/19^−^ cells combined with forward scatter (FSC)/ side scatter (SSC) characteristics and CD45 expression. The gating strategy used is shown in Fig. S1. Antibodies used included anti-human CD160-Alexa Fluor 488, CD4-APC-Fire750, CD8-BV510, HLA-DR-Alexa Fluor 700, CD14-APC, PD-1-PE, 2B4-PE-CF594, CD16-BV711, TIM-3-BV650, CD200R-PE, BTLA-BV650, CD45-BV786 (BD Biosciences, San Diego, CA, USA), CX3CR1-BV421, CD3-PerCP-Cy5.5, CD15-PerCP-Cy5.5, CD19-PerCP-Cy5.5, CD29-Alexa Fluor 488, CD62L-BV650, CD11b-BV605, CCR2-PE (BioLegend, San Diego, CA, USA), TIGIT-PE-Cy7, and LAG-3-APC (eBioscience, San Diego, CA, USA), along with the corresponding isotype controls. BD Trucount Tubes (BD Biosciences), combined with specific antibodies (CD45/3/4/8 cocktail; BD Biosciences), were used to determine the absolute counts of leukocytes in the blood with flow cytometry according to the manufacturer’s instructions. The absolute numbers (cells per microliter) of leukocytes and T cells were determined by comparing cellular and bead events.

### In vitro stimulation and intracellular staining

PBMCs were cultured in RPMI-1640 media (GIBCO, Grand Island, NY, USA) containing 10% fetal bovine serum (FBS), with or without LPS (100 ng/mL, STEMCELL Technologies, Vancouver, Canada) and Golgiplug (BD Biosciences, San Diego, CA, USA) for 3 h. The cells were surface-stained with CD45-BV786, CD14-Alexa Fluor 700, CD16-BV711, HLA-DR-APC-H7, TIM-3-BB515 (BD Biosciences, San Diego, CA, USA), and TIGIT-PE-Cy7 (eBioscience, San Diego, CA, USA), and intracellularly stained with antibodies against IL-10-APC, IL-1β-Pacific blue, TNF-α-BV650 (BD Biosciences), IL-6-PE (eBioscience), GM-CSF-PE-CF594 (BioLegend), and the corresponding isotype controls. Data acquisition was performed on an LSR Fortessa flow cytometer (BD Biosciences, San Diego, CA, USA), and data were analyzed with FlowJo software (Tree Star, Ashland, OR, USA).

### Statistical analysis

Data are expressed as the mean ± standard deviation (SD). GraphPad Prism 7 (GraphPad Software, La Jolla, CA, USA), SPSS (IBM Corporation, New York, NY, USA), and the R program (https://cran.r-project.org/) were used for statistical calculations. The normality of each variable was evaluated using the Kolmogorov–Smirnov test. For comparing two or more independent samples, a Kruskal–Wallis test followed by Dunn’s multiple comparisons test was used. Participant characteristics were compared using the Kruskal–Wallis ANOVA test (continuous variables), followed by post hoc Bonferroni analyses. If the data were not symmetric, the Greenhouse–Geisser correction was used. For all analyses, *P*-values < 0.05 were considered statistically significant.

## Supplementary Information

**Additional file 1.**

**Additional file 2.** 

**Additional file 3.**

## Data Availability

Not applicable.

## Abbreviations

PBMCs: Peripheral blood mononuclear cells
TNF-α: Tumor necrosis factor-α
IL-10: Interleukin-10
GM-CSF: Granulocyte-macrophage colony-stimulating factor
IL-1β: Interleukin-1β
HLA-DR: Human leukocyte antigen-DR
CD11b: Adhesion molecule cluster of differentiation molecule 11b
CD62L: L-selectin
CX3CR1: C-X-C chemokine receptor
PD-1: Programmed death-1
TIM-3: T-cell immunoglobulin domain and mucin domain 3
LAG-3: Lymphocyte-activation gene 3
BTLA: B and T lymphocyte attenuator
TIGIT: T-cell immunoglobulin and immunoreceptor tyrosine-based inhibitory motif (ITIM) domain
PBS: Phosphate-buffered saline
LPS: Lipopolysaccharide
MFI: Median fluorescent intensity
SD: Standard deviation
