# Supplementary material for: Phenotypic and functional alterations of monocyte subsets with aging
Source: Immun Ageing. 2022 Dec 13;19:63. doi: 10.1186/s12979-022-00321-9 (PMC9745938; doi:10.1186/s12979-022-00321-9)
Supplement: Supplementary file 1 — Additional file 1. [file 12979_2022_321_MOESM1_ESM.pdf]

Figure S1. Gating strategy of monocyte subsets and the number of total monocyte and monocyte subsets among young, middle-aged, and older adults.

A. The fresh isolated PBMCs were gated for forward and side-scatter (FSC/SSC), doublets, live/dead and $CD45^{+}CD3^{-}CD15^{-}CD19^{-}$ prior to identification of monocytes.

B. Comparison of the number of total monocytes among whole blood in young, middle-aged, and older adults (young: 21-40 years, n = 42; middle-aged: 41-60 years, n = 34; older: >60 years, n = 34). *P*-values were calculated using the non-parametric Kruskal-Wallis rank test and followed by post hoc analysis.

C. Comparison of the number of Mo0, Mo1, Mo2 and Mo3 subpopulations among all monocytes in young, middle-aged, and older adults were performed according to the expression pattern of CD14 and CD16 (young: 21-40 years, n = 42; middle-aged: 41-60 years, n = 34; older: >60 years, n = 34). *P*-values were calculated using the non-parametric Kruskal-Wallis rank test and followed by post hoc analysis.

Figure S2. Representative flow cytometry data of cytokine-secreting capacity of total monocytes with mock controls or LPS stimulation.

A. The TNF-α and IL-6 producing capacity of total monocyte with mock controls and LPS stimulation (100ng/ml) for 3h *in vitro* from young adults.

B. The TNF-α, IL-6, IL-10, GM-CSF and IL-1β producing capacity of total monocyte with mock controls from young, middle-aged and older adults (young:21-40 years,

n = 9; middle-aged:41-60 years, n = 9; older: >60 years, n = 15). *P*-values were obtained by a Kruskal-Wallis test and followed by post hoc analysis.

Figure S3. The percentage and median fluorescent intensity (MFI) of total monocyte cytokines from young, middle-aged and older adults

A. Intracellular staining for IL-10, GM-CSF and IL-1β in total monocytes from young, middle-aged and older adults (young:21-40 years, n = 42; middle-aged:41-60 years, n = 34; older: >60 years, n = 34) upon *in vitro* LPS stimulation. *P*-values were obtained by a Kruskal-Wallis test and followed by post hoc analysis.

B. The MFI of IL-10, GM-CSF, IL-1β, TNF-α and IL-6 on total monocyte from young, middle-aged and older adults (young:21-40 years, n = 42; middle-aged:41-60 years, n = 34; older: >60 years, n = 34) upon *in vitro* LPS stimulation. *P*-values were obtained by a Kruskal-Wallis test and followed by post hoc analysis.

Figure S4. The MFI of HLA-DR, CD88, CD29, CD11b and CD62L on monocyte subsets.

A. The histograms of CD11b, HLA-DR, CD62L, CD29, and CD88 on monocyte subsets from young adults.

B. The MFI of CD11b, HLA-DR, CD62L, CD29, and CD88 on monocyte subsets from young, middle-aged and older adults.

Figure S5. The MFI of CD29 and CD88 on monocyte subsets from young, middle-

aged and older adults.

A. Line plot demonstrating the dynamic trend profiling of the MFI of HLA-DR, CD11b, CD62L, CD29, CD88 (loess smoothed normalized counts ± standard error (SE)) from monocyte subsets in 21-40,41-60, >60 three groups healthy individuals. (young:21-40 years, n = 23; middle-aged:41-60 years, n = 15; older: >60 years, n = 44)

B. The boxplots show that the MFI of CD29 and CD88 on monocyte subsets from young, middle-aged and older adults. *P*-values were obtained by a Kruskal-Wallis test and followed by post hoc analysis.

Figure S6. The MFI of CCR2 and CX3CR1 on monocyte subsets from young, middle-aged and elderly adults.

A. The histograms of CCR2 and CX3CR1 from monocyte subsets in 21-40, 41-60, >60 three groups.

B. Line plot demonstrating the dynamic trend profiling of the MFI of CCR2 and CX3CR1(loess smoothed normalized counts ± standard error (SE)) from monocyte subsets in 21-40, 41-60, >60 three groups. (young:21-40 years, n = 23; middle-aged:41-60 years, n = 15; older: >60 years, n = 44)
