# Supplementary figures and images for: Phenotypic and functional alterations of monocyte subsets with aging
Source: Immun Ageing. 2022 Dec 13;19:63. doi: 10.1186/s12979-022-00321-9 (PMC9745938; doi:10.1186/s12979-022-00321-9)

A

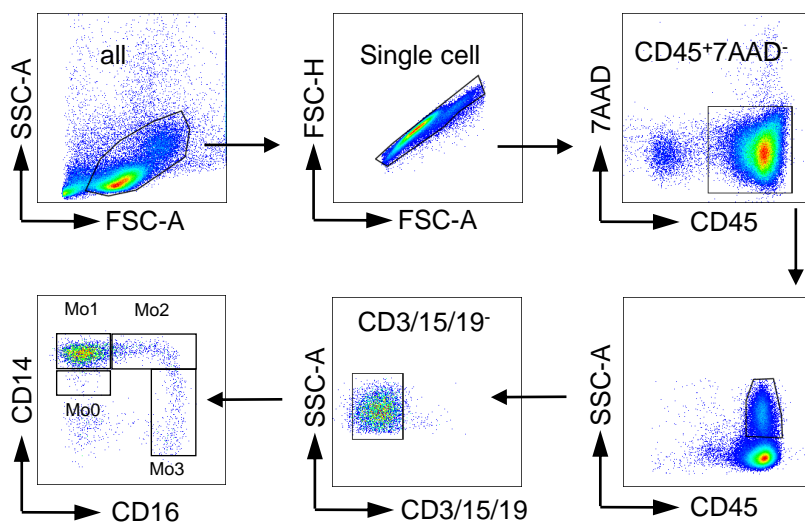

B

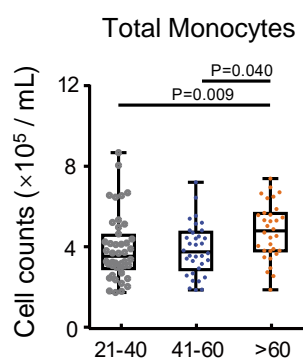

C

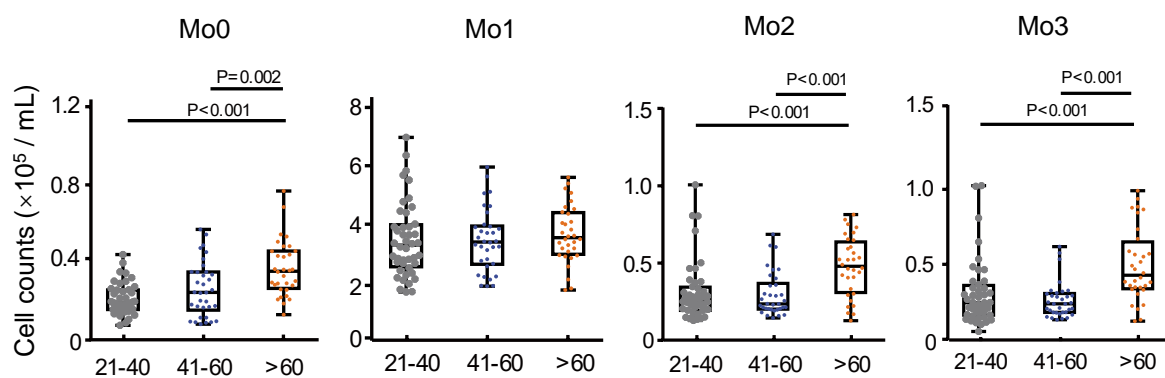

Fig. S1

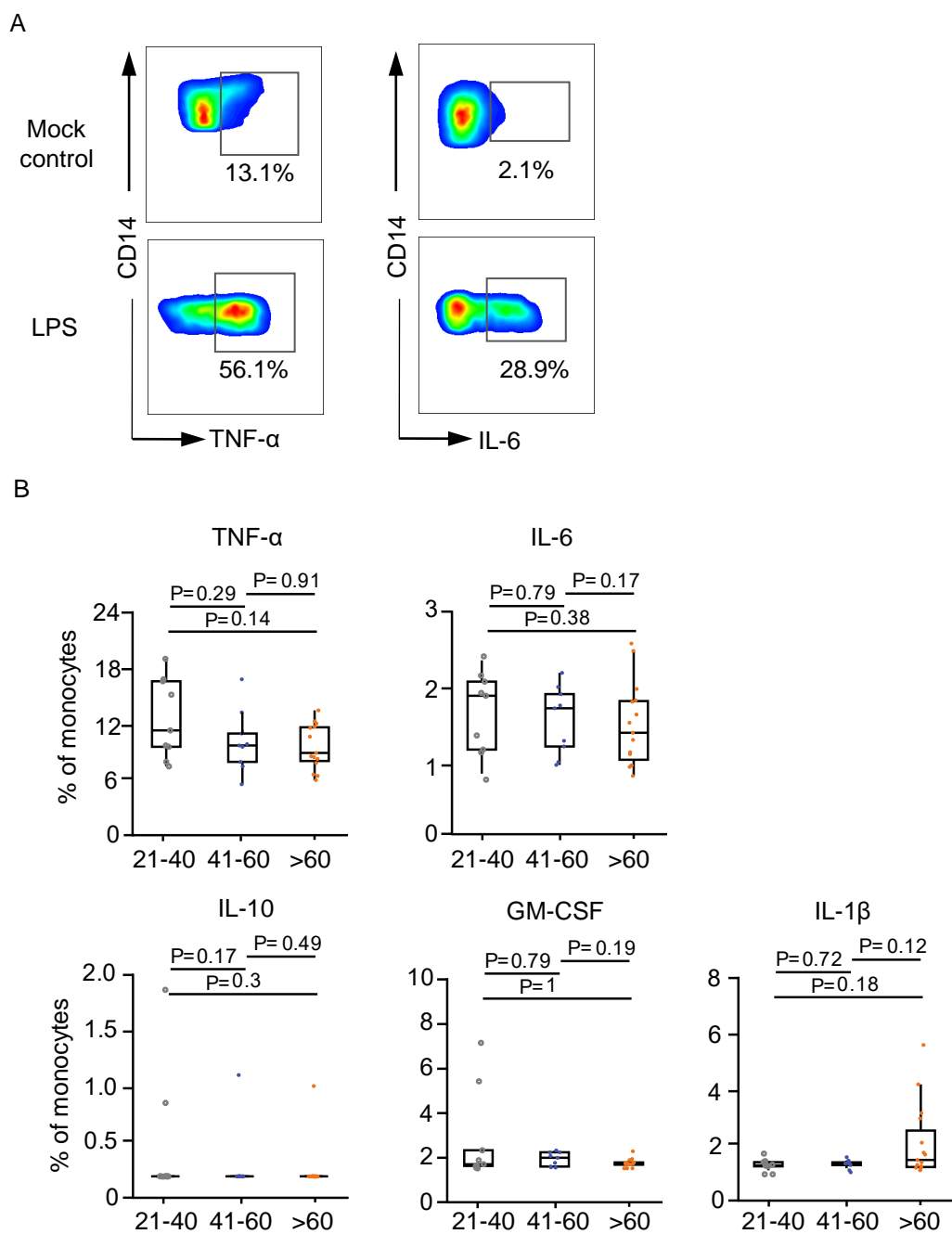

Fig. S2

A

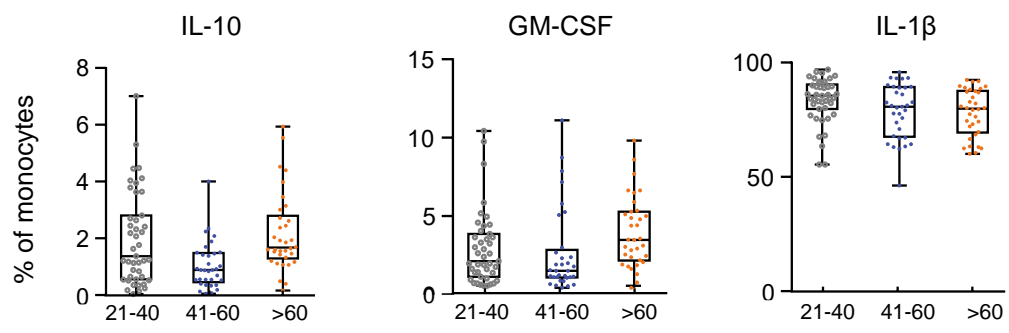

B

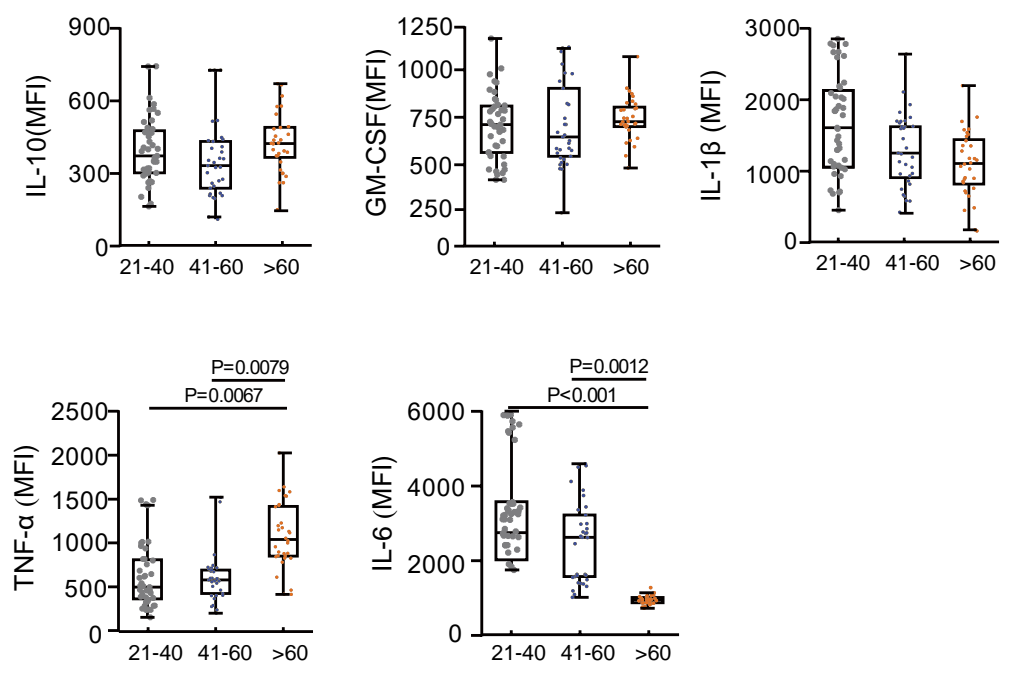

Fig. S3

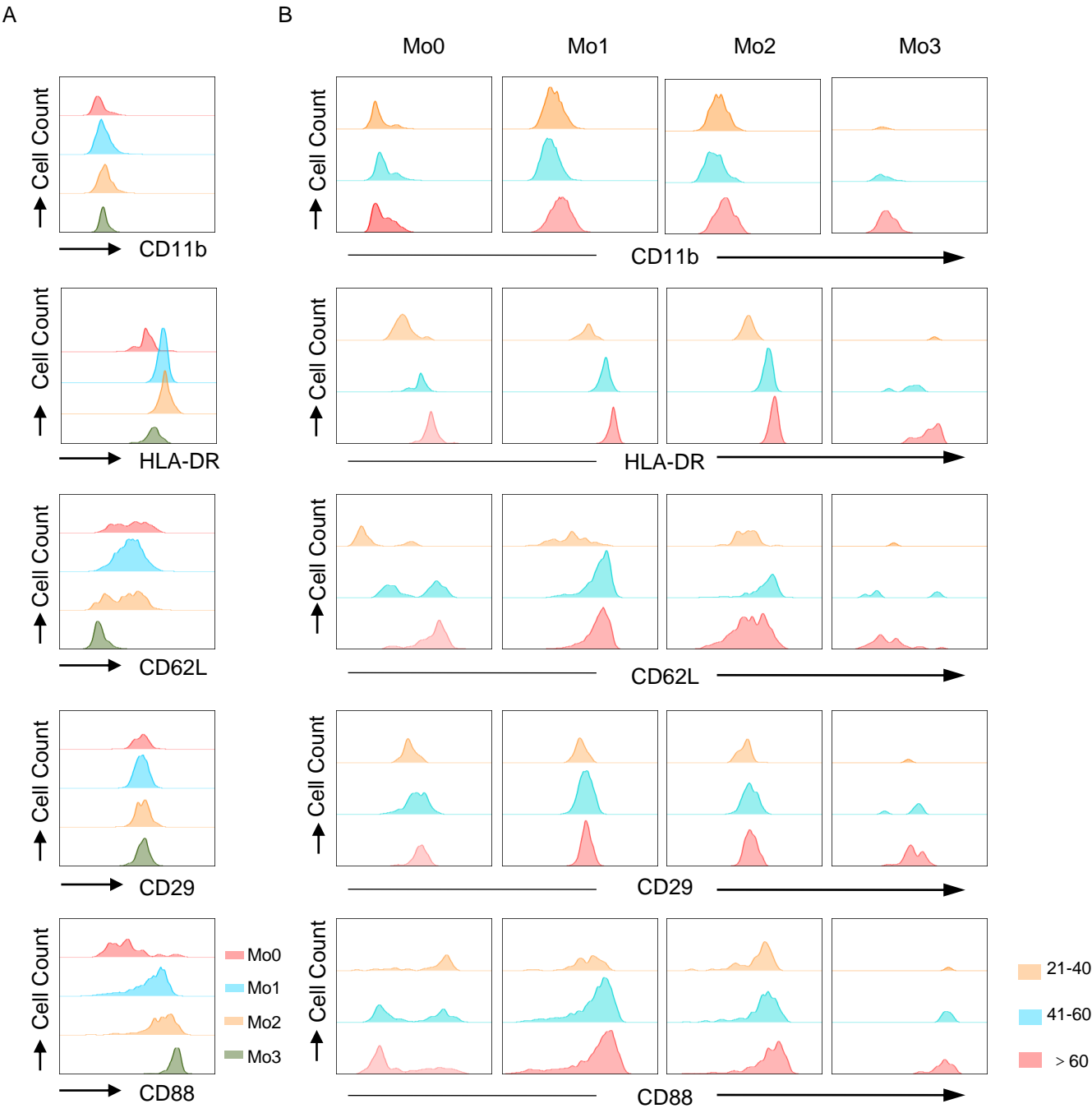

Fig.S4

A

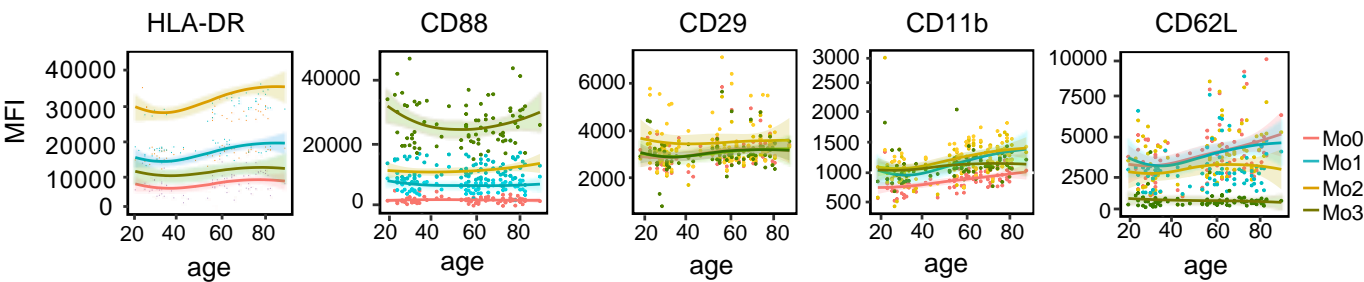

B

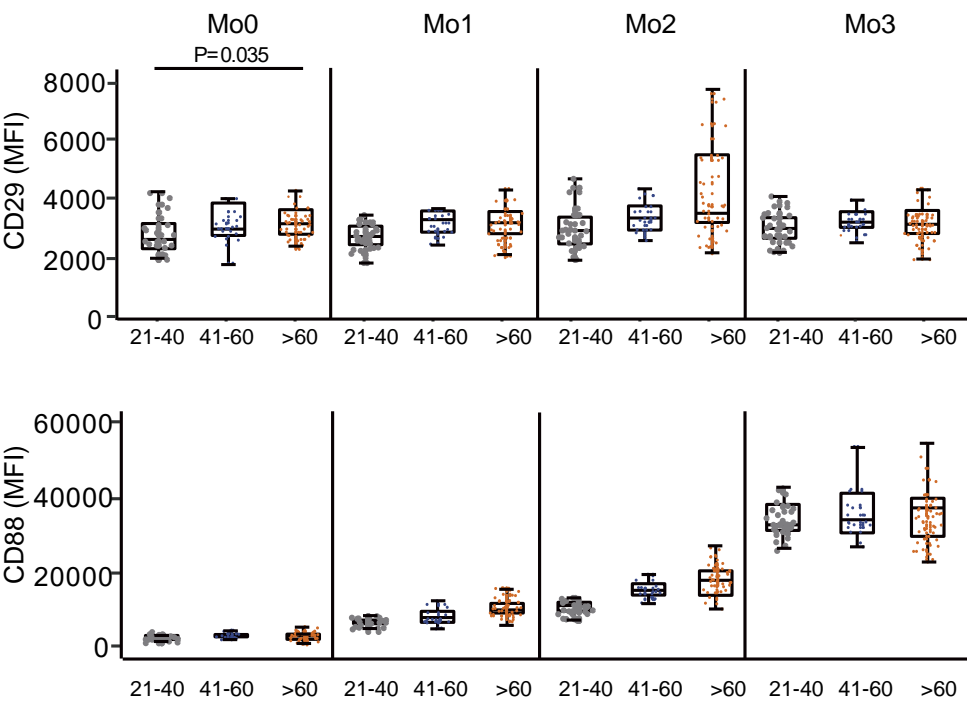

Fig.S5

A

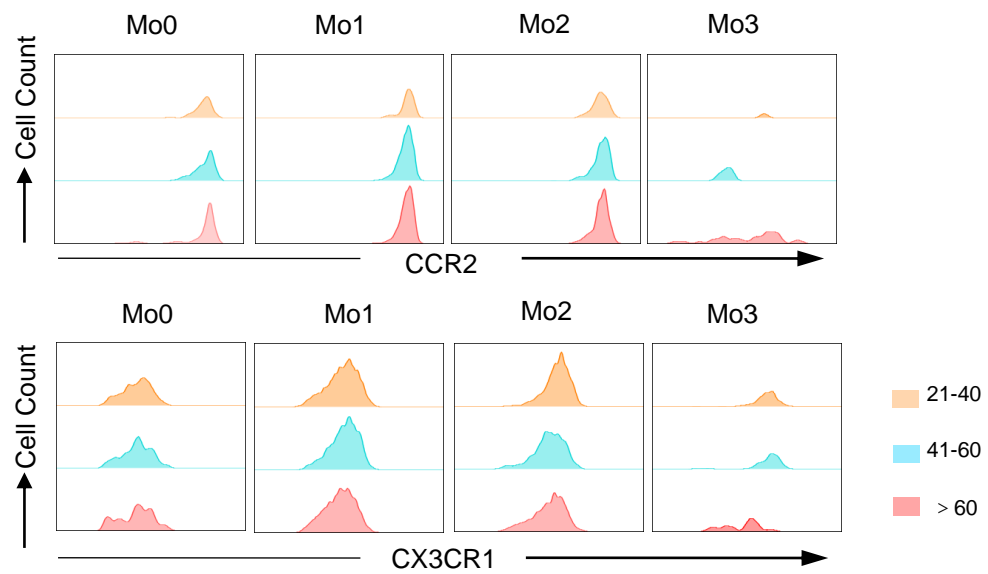

B

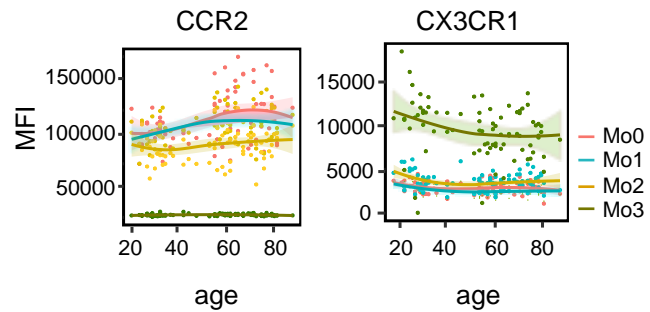

Fig.S6

Supplement: Supplementary file 2 — Additional file 2. [file 12979_2022_321_MOESM2_ESM.pdf]
