# Supplementary material for: Phenotypic and functional alterations of monocyte subsets with aging
Source: Immun Ageing. 2022 Dec 13;19:63. doi: 10.1186/s12979-022-00321-9 (PMC9745938; doi:10.1186/s12979-022-00321-9)
Supplement: Supplementary file 3 — Additional file 3. [file 12979_2022_321_MOESM3_ESM.docx]

|  | Markers | Function | Reference |
| --- | --- | --- | --- |
| Adhesion  molecules  receptors | CD11b | Mediated the trans-endothelial migration of monocytes | 50 |
|  | CD62L | Involved in monocyte rolling and adhesion to endothelial cells | 52 |
|  | CD29 | Intercellular adhesion | 55 |
| Activation molecules  receptors | HLA-DR | A major histocompatibility complex (MHC) class II molecule | 41,43 |
|  | CD88 | The C5a-receptor of chemotactic and inflammatory peptide anaphylatoxin | 42 |
| Chemokine receptors | CCR2 | The receptor for MCP-1/CCL2 mediating enhanced chemotactic motility and recruitment of cells to the vessel wall | 51 |
|  | CX3CR1 | The receptor for fractalkine/CX3CL1 of providing the survival signal and promotes the differentiation of non-classical monocytes into anti-inflammatory M2-like macrophages | 22 |
| Co-inhibitory molecules | BTLA | Impairs TNF-α production by negatively regulating the NF-κB/TNF-α pathway | 46 |
|  | 2B4 |  | 47 |
|  | TIM-3 |  | 45,46 |
|  | CD200R |  | 47 |
|  | TIGIT | Related to T cells exhaustion | 10 |
|  | PD-1 |  | 10 |
|  | CD160 |  | 12 |
|  | LAG-3 |  | 12 |

Table S1 Molecules were selected to characterize the immune status of monocyte populations.
